# An advanced pulmonary sarcomatoid carcinoma patient harboring a BRAF^V600E^ mutation responds to dabrafenib and trametinib: a case report and literature review

**DOI:** 10.3389/fonc.2023.1220745

**Published:** 2023-07-21

**Authors:** Ruoxin Fang, Jun Gong, Zhengkai Liao

**Affiliations:** Department of Radiation and Medical Oncology, Zhongnan Hospital of Wuhan University, Hubei Key Laboratory of Tumor Biological Behaviors, Hubei Cancer Clinical Study Center, Wuhan, Hubei, China

**Keywords:** pulmonary sarcomatoid carcinoma, BRAF V600E mutation, dabrafenib, trametinib, case report

## Abstract

**Background:**

The pulmonary sarcomatoid carcinoma (PSC) is a rare and aggressive subtype of NSCLC with rapid progression and poor prognosis, and is resistant to conventional chemotherapy. Most PSC cases have potential targetable genomic alterations. Approximately 7% of PSC patients have BRAF mutations, and the efficacy of dabrafenib and trametinib in BRAF^V600E^ mutated PSC is unclear.

**Case presentation:**

Our report describes a patient with mutated BRAF^V600E^ PSC who underwent surgery and adjuvant chemotherapy early but quickly relapsed. Both chemotherapy and immunotherapy were ineffective for him, combined dabrafenib and trametinib produced a 6-month progression-free survival, and a partial response was observed in the tumor response evaluation. As a result of financial pressure, he stopped taking the targeted drugs, and his disease rapidly progressed.

**Conclusion:**

Dabrafenib combined with trametinib provides partial remission in patients with advanced PSC with BRAF^V600E^ mutations, and large-scale NGS panels could offer more options for PSC treatment.

## Introduction

In less than 1% of all lung cancers, pulmonary sarcomatoid carcinoma (PSC) is a rare subtype of non-small cell lung cancer (NSCLC), with high malignancy and poor prognosis (1). Despite early diagnosis, PSC patients have a worse prognosis compared to other types of NSCLC (2). According to the analyzes of the National Cancer Data Base, the 5-year survival rate for PSC was only 14.4% and the median overall survival (OS) was only 6.4 months (3). Because PSC is greatly resistant to conventional first-line chemotherapy drugs (4), there is an urgent need to actively explore the multimodal treatment of PSC. Previous studies have shown that most PSC cases have potential targetable genomic alterations and intermediate or high tumor mutation burden (TMB) (5). One study showed that patients with PSC treated with chemotherapy combined with immunotherapy as first-line therapy achieved an objective response rate (ORR) of 73.8% and a median progression-free survival (mPFS) of 10.3 months (6). There are also some case reports that the use of targeted drugs significantly alleviates disease and even survival benefits in patients with PSC (7–9). Thus, immunotherapy and targeted therapy may offer new opportunities for the treatment of PSC.

BRAF, a member of the Ras/mitogen-activated protein kinase signaling pathway, is downstream of KRAS, and directly phosphorylates MEK, which in turn phosphorylates ERK. The pathway culminates in the transcription of genes favoring proliferation and survival (10). The mutation frequency of BRAF in PSC is 7.2% (5). Moreover, the most common BRAF mutation, V600E (Val600Glu), was observed in 1–2% of lung adenocarcinomas (11). The combination of dabrafenib and trametinib is safe and effective in NSCLC patients with BRAF^V600E^ mutations (12–14). However, existing studies have not reported the efficacy of this combination in patients with PSC. Here, we report a patient with BRAF^V600E^ mutated-PSC who underwent surgery and postoperative adjuvant chemotherapy at an early stage but quickly relapsed. After relapsing, he received chemoradiotherapy in combination with immunotherapy and continued to progress rapidly, after which he achieved six months of PFS with dabrafenib in combination with trametinib. Despite his financial difficulties, he stopped using targeted drugs and switched to immunotherapy, after which his disease progressed rapidly.

## Case presentation

A 67-year-old man, with a 30-year history of smoking and a five-year history of hypertension, came to Zhongnan Hospital of Wuhan University with a cough on February 1, 2021. Since the nodule of the left lower lobe did not change significantly (**Figure S1**), the attending doctor ignored it. The patient’s cough did not abate and he went to another hospital on April 8, 2021. A PET-CT examination revealed a nodule in the basal segment of the left inferior lobe with cavity and increased metabolism. Then, he underwent a lower left lobectomy and lymph node dissection on April 15. The pathological findings of the excised tissue suggested PSC (d=1.3cm) and no lymph node metastasis. In addition, the tumor stage was T1aN0M0. Considering the elevated risk of postoperative recurrence and poor prognosis of PSC, he then received four cycles of postoperative adjuvant chemotherapy (gemcitabine 2200 mg d1, d8 + cisplatin 40 mg d1~d3) from June 3 through August 6 in another hospital, during which severe myelosuppression and anemia occurred, and reexamination showed no tumor recurrence.

**Figure 1:**
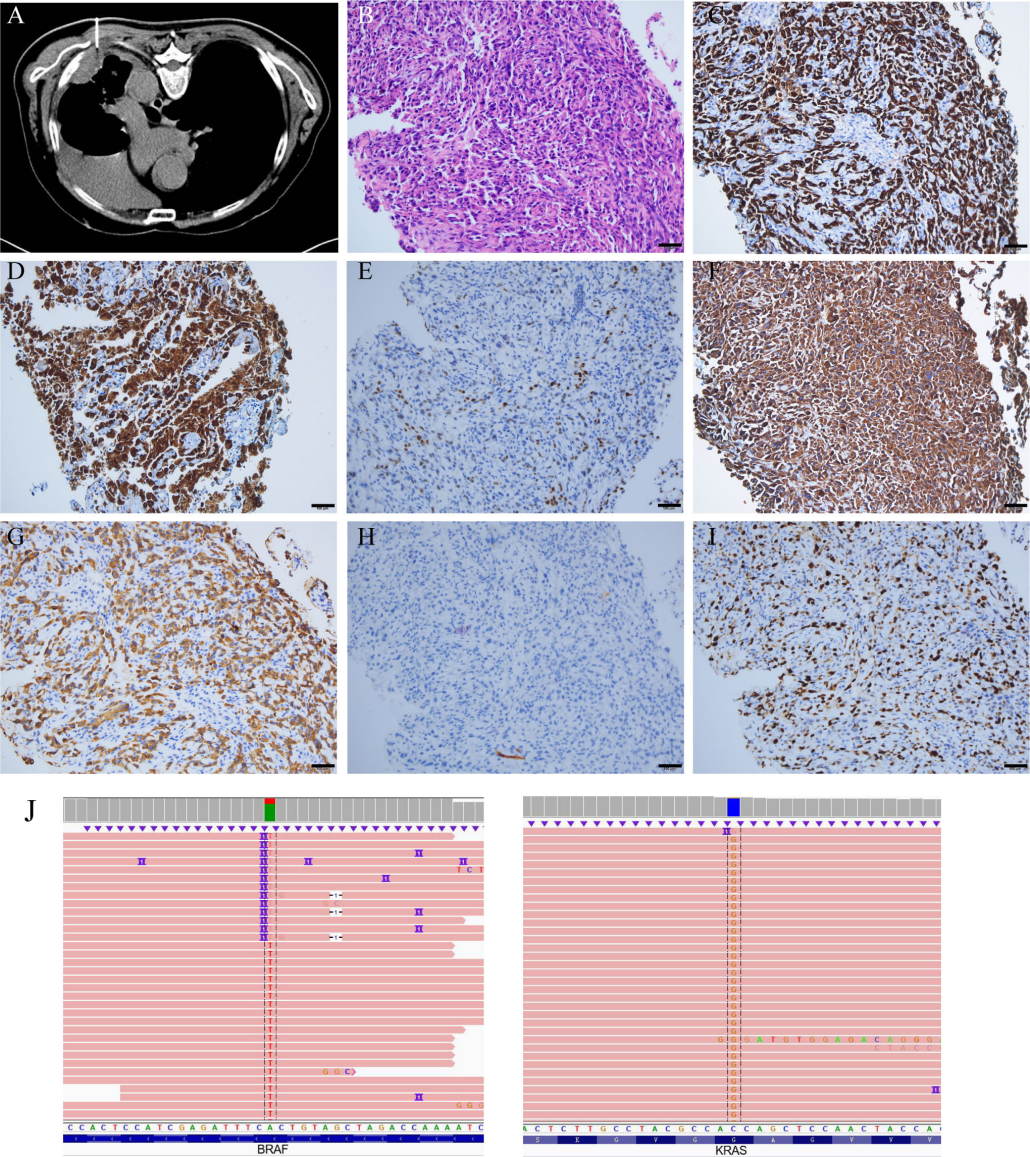
CT-guided needle biopsy, and its pathological staining and NGS analysis. **(A)** CT-guided needle biopsy. **(B)** HE staining, spindle cells, scale bars: 100μm. **(C)** CK7 (+). **(D)** NapsinA (focal +). **(E)** TTF-1 (partial +). **(F)** VIMENTIN (spindle cell +). **(G)** CK (+). **(H)** P40 (-). **(I)** Ki-67 (50%). **(J)** NGS revealed the presence of the BRAF^V600E^ mutation (exon 15, 22.7% abundance) and KRAS^G12A^ mutation (exon 2, 5.71% abundance). Technical details are provided in the **Supplementary Material S5**.

**Figure 2:**
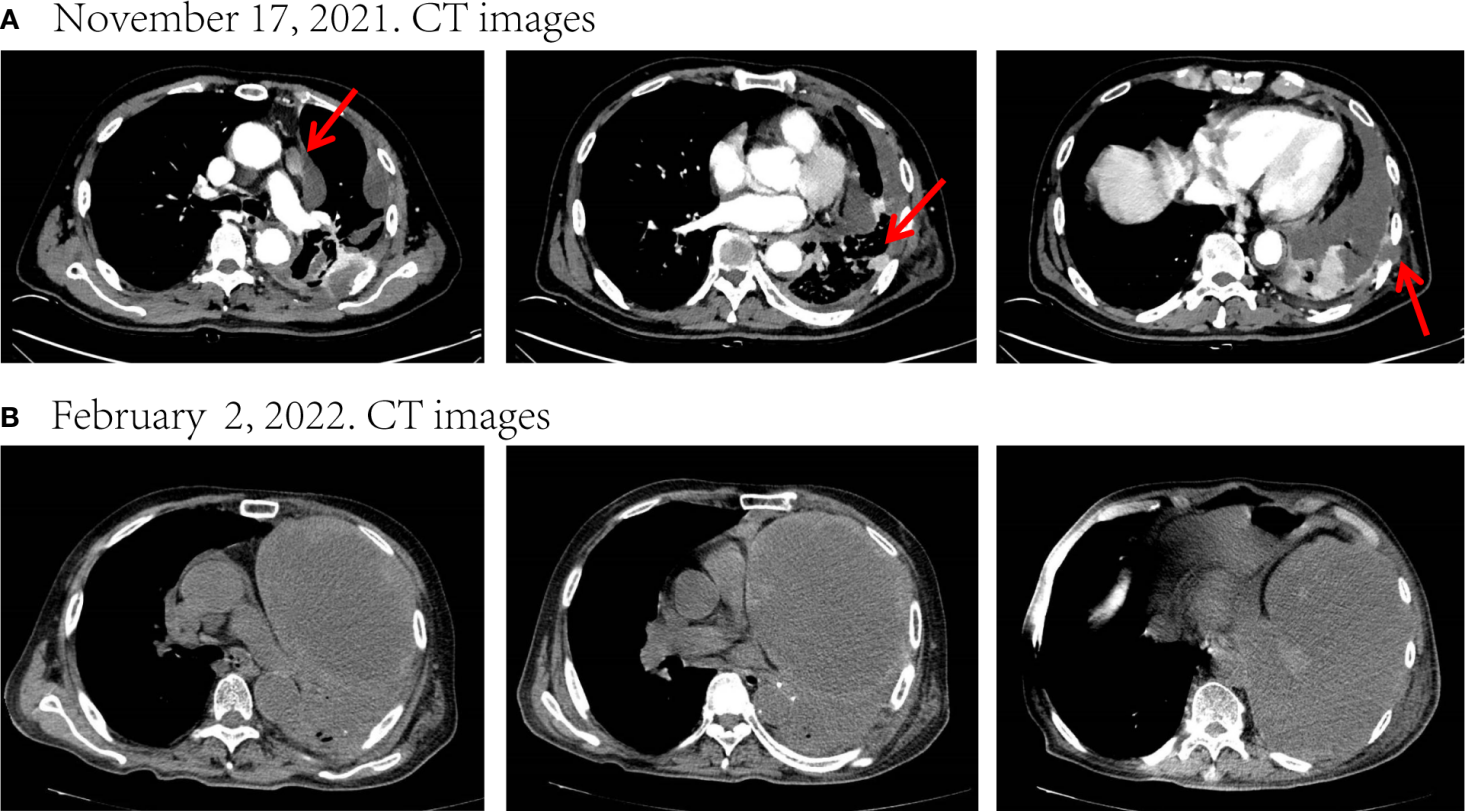
Disease status after chemoradiotherapy and two-cycle immunotherapy. **(A)** CT images before treatment. **(B)** After chemoradiotherapy and two-cycle immunotherapy, there was a marked increase in the size of the mass and a massive encapsulated malignant pleural effusion leading to left atelectasis. The tumor response evaluation is a progressive disease (PD). Red arrows indicate tumor or lymph node lesions.

However, on October 21, 2021, he came to Zhongnan Hospital of Wuhan University again due to fatigue and chest pain, and out-patient CT showed enlarged nodules of left lung and subpleural, enlarged mediastinal lymph nodes and multiple low-density shadows on left ribs (**Figure S2**). To clarify the histology of the mass, a lung needle biopsy was performed on November 16 (**Figure 1A**) and an enhanced CT was reviewed (**Figure 2A**). Microscopically, two morphologies of cells, spindle cells, and epithelial cells were seen (**Figure 1B**). The immunohistochemical results were as follows: CK7 (+), NapsinA (focal +), TTF-1 (partial +), VIMENTIN (spindle cell +), CK (+), P40 (-), Ki-67 (50%) (**Figures 1C–I**), CK5/6 (-), SYN (-), CD56 (-), CgA (-). PD-L1 combined positive score (CPS) of the small amount of tissue taken for biopsy was 0%. The above results support the diagnosis of lung adenocarcinoma poorly differentiated with spindle cell components (15). Combined with the pathological diagnosis of the original surgical specimen (**Figure S3**), it was consistent with the involvement of PSC. Based on the clinical information, we considered it was a PSC recurrence. Upon next-generation sequencing (NGS) analysis of the biopsy tissue sample, the patient was identified with BRAF^V600E^ mutation (exon 15, 22.7% abundance), KRAS^G12A^ mutation (exon 2, 5.71% abundance) (**Figure 1J**), PIK3CA^E707K^ mutation (exon 14, 1.02% abundance) and TP53^H179R^ mutation (exon 5, 29.65% abundance).

To relieve the chest pain, he began to receive radiotherapy (Dt=45Gy/15F) on November 29 for the destruction of the ribs and the surrounding carcinoma tissue (**Figure S4**). After radiotherapy, his chest pain eased significantly. He refused targeted therapy for financial reasons. Later, he received one-cycle chemotherapy (albumin-bound paclitaxel 400mg d1 + carboplatin 400mg d1) in combination with tislelizumab (200mg d1) on December 18 and was pleural infused with cisplatin (40mg) on December 20. Unfortunately, he developed severe post-chemotherapy myelosuppression, malnutrition, and pulmonary infection, as well as a large pleural effusion, and the family refused to perform pleurocentesis. After symptomatic treatment, the patient continued to be unable to tolerate chemotherapy, so he was only treated with tislelizumab (200mg d1) on January 22, 2022. However, his condition continued to deteriorate. On February 2, he was re-admitted to the hospital due to fever and dyspnea. CT showed that the left lung was atelectatic due to a tumor and massive pleural effusion (**Figure 2B**), and the Eastern Cooperative Oncology Group (ECOG) score was up to 3. He could no longer tolerate chemotherapy and immunotherapy, and after adequate communication, he opted for targeted therapy with oral dabrafenib and trametinib.

**Figure 3:**
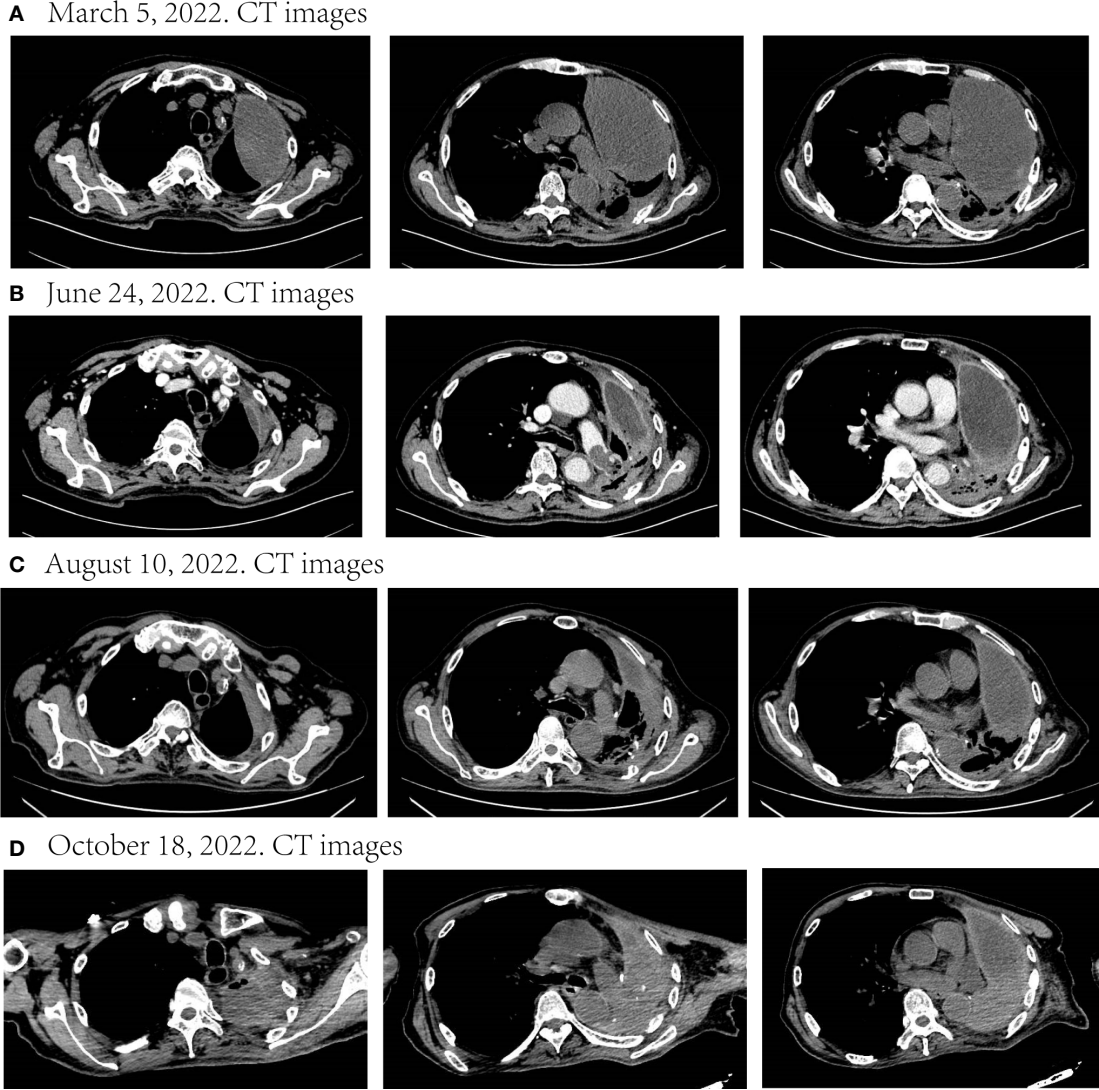
Disease status after targeted therapy and palliative immunotherapy. **(A–C)** During nearly six months of targeted therapy, the malignant pleural effusion continued to decrease and the mass continued to shrink. The tumor response evaluation of the three times respectively was SD, PR, and SD. **(D)** After discontinuation of targeted therapy and palliation with immunotherapy, the disease progressed. SD, stable disease; PR, partial response.

Although he suffered adverse reactions such as pruritus and loss of appetite during the course of the targeted medication, he improved after treatment. Meanwhile, his reexamination results showed that malignant pleural effusion was significantly reduced and the mass significantly retreated (**Figures 3A–C**). The tumor response evaluation reached partial response (PR). Nonetheless, he stopped taking dabrafenib and trametinib in July 2022 because of financial pressures and began palliative antitumor therapy with tislelizumab (200mg d1) on August 12. His last imaging examination revealed strict left lung atelectasis, increased malignant pleural effusion, and mass enlargement (**Figure 3D**). Through follow-up, we learned that he passed away at home on November 7, 2022.

## Discussion

PSC is a rare and aggressive subtype of NSCLC that progresses rapidly, has a poor prognosis, and is resistant to conventional chemotherapy. The incidence of PSC is less than 1% of all primary lung tumors (16). Based on histological features, PSC can be classified as pleomorphic carcinoma, pulmonary blastoma, and carcinosarcoma. Pleomorphic carcinoma is the most common and can be divided into giant cell carcinoma and spindle cell carcinoma (15). The occurrence of PSC, especially pleomorphic carcinoma, may be related to epithelial-mesenchymal transition (EMT). Some case reports have confirmed the transformation of lung squamous cell carcinoma or lung adenocarcinoma into PSC during treatment, confirming the EMT process (17, 18). PSC has a poor prognosis due to its difficulty in early diagnosis, insensitivity to conventional treatment, and poor biological behavior (4). Chen et al. analyzed the cases in the SEER database and found that the OS of PSC was still much lower than other types of NSCLC after balancing different characteristics (19). In previous SEER-based studies, the median OS (mOS) was 6.0 months for PSC patients with all stages and 3.0 months for patients with advanced disease (20, 21). Currently, most people with PSC are still treated according to the standard treatment for NSCLC, and there is an urgent need to explore effective treatments for PSC.

For patients with PSC at an early stage, surgery is the best option (22, 23). However, surgeons are seldom asked to make management decisions regarding PSC (24), and there is still debate about whether adjuvant chemotherapy can benefit patients with PSC. A study based on the SEER database concluded that adjuvant chemotherapy did not significantly benefit OS in patients with PSC (25). While Abdallah et al. through the analysis of the National Cancer Database found that adjuvant chemotherapy appears to be associated with improved survival in stage II and stage III, but not stage I patients (26). In the study of Maneenil et al., 33 out of 45 patients who underwent complete resection had a definite recurrence (27). To reduce the likelihood of recurrence, the patient in our report received adjuvant chemotherapy despite being stage I at the time of surgery. In terms of the choice of chemotherapy regimen, previous studies have shown that PSC is insensitive to most chemotherapy drugs (28), but platinum-based combination chemotherapy can improve OS, with mOS of 3.95 months in patients receiving platinum-based chemotherapy, while only 0.95 months in patients who do not receive (29). Therefore, platinum-based combination chemotherapy remains a better option for PSC compared to alternative chemotherapy regimens.

Currently, immunotherapy has shown increasing potential for PSC treatment. Immune-checkpoint inhibitors (ICIs) achieve the purpose of anti-tumor by enhancing the activity of CD4^+^ and CD8^+^ T lymphocytes, which can improve the clinical prognosis of various tumors. The KEYNOTE-189 trial showed that for patients with metastatic nonsquamous NSCLC without sensitizing EGFR or ALK mutations, regardless of the patient’s programmed cell death-ligand 1 (PD-L1) expression, even <1%, the use of chemotherapy in combination with immunotherapy as first-line treatment can significantly improve the survival time of patients (30, 31). The RATIONALE-307 trial showed that tislelizumab combined with paclitaxel and carboplatin significantly improved PFS and ORR in patients with advanced squamous NSCLC, regardless of PD-L1 expression (32). ICIs have been used as adjuvant therapy for patients with advanced lung cancer (4). About 40.6% of Chinese patients with PSC were shown to have high TMB (33), and most PSC patients had significantly higher PD-L1 expression than NSCLC patients (34), suggesting that PSC patients may benefit from ICIs treatment. According to one case report, a patient of PSC with low expression of PD-L1 showed a remarkable response to pembrolizumab plus cytotoxic chemotherapy as the first-line treatment (35). In a cohort study of 42 PSC patients treated with ICIs, the ORR of the overall population was 73.8%, regardless of PD-L1 expression status, suggesting that chemotherapy combined with immunotherapy has a significant efficacy as the first-line treatment for PSC patients (6). Thus, while CPS=0 for PD-L1 in this patient, immunotherapy could theoretically benefit him.

Whereas neither chemotherapy nor immunotherapy resulted in remission in this patient we report on, only targeted therapy has benefited him. Previous studies have shown that most PSC cases have potential targetable genomic alterations (5). One of the most popular is the mesenchymal to epithelial transition (MET) proto-oncogene alterations, and METex14 skipping mutation is the most common and well-studied mutation type, occurring in about 22%~31.8% of PSC patients (36, 37). A phase II study showed savolitinib in Chinese patients with PSC and other NSCLCs harboring METex14 skipping mutation with an ORR of 49.2% (38). There are still numerous potential therapeutic targets in PSC that are worth exploring. Yang et al. performed whole exome sequencing on 56 PSC samples, of which TP53 mutations were the most common, accounting for 79% of the entire cohort. 57% of the patients harbored mutations in genes of the receptor tyrosine kinase/RAS pathway: 14% carried KRAS mutations, and 7% carried BRAF mutations. Two of the four patients with the BRAF mutation had the V600E mutation (39). In addition, a recent study demonstrated that BRAF mutation did not modulate the NSCLC response to ICIs (40). This evidence suggests that BRAF mutations are not a disadvantage to the efficacy of ICIs. V600E is the most common type of BRAF mutations, accounting for about 1-2% of lung adenocarcinomas (11). Dabrafenib and trametinib inhibits BRAF and MEK, and in its phase II trial, demonstrated a response of 67% and duration of response of 9.8 months in patients with previously treated BRAF^V600E^-mutant metastatic NSCLC (13, 14), a response of 64% and a median response duration of 10.4 months in patients with previously untreated NSCLC (12). The National Medical Products Administration (NMPA) approved dabrafenib in combination with trametinib for the treatment of BRAF^V600E^ mutated NSCLC in China on March 24, 2022. However, no PSC patients were included in these cohorts, and the efficacy of dabrafenib in combination with trametinib in PSC patients with BRAF^V600E^ mutations is unclear. As a result, the patient was not treated with targeted drugs as soon as the genetic test results were clear. Moreover, in China, treatment with dabrafenib combined with trametinib costs about 22,000 yuan (3,157 $) per month at that time, much higher than the average monthly income of Chinese people (3,074 yuan, 448 $), which causes a serious financial burden for an average family. Therefore, the patient eventually gave up on continuing to take targeted drugs. The rapid progression of the disease in this patient despite subsequent maintenance with tislelizumab is evidence from another perspective that dabrafenib combined with trametinib effectively controls tumor progression in this patient.

**Figure 4:**
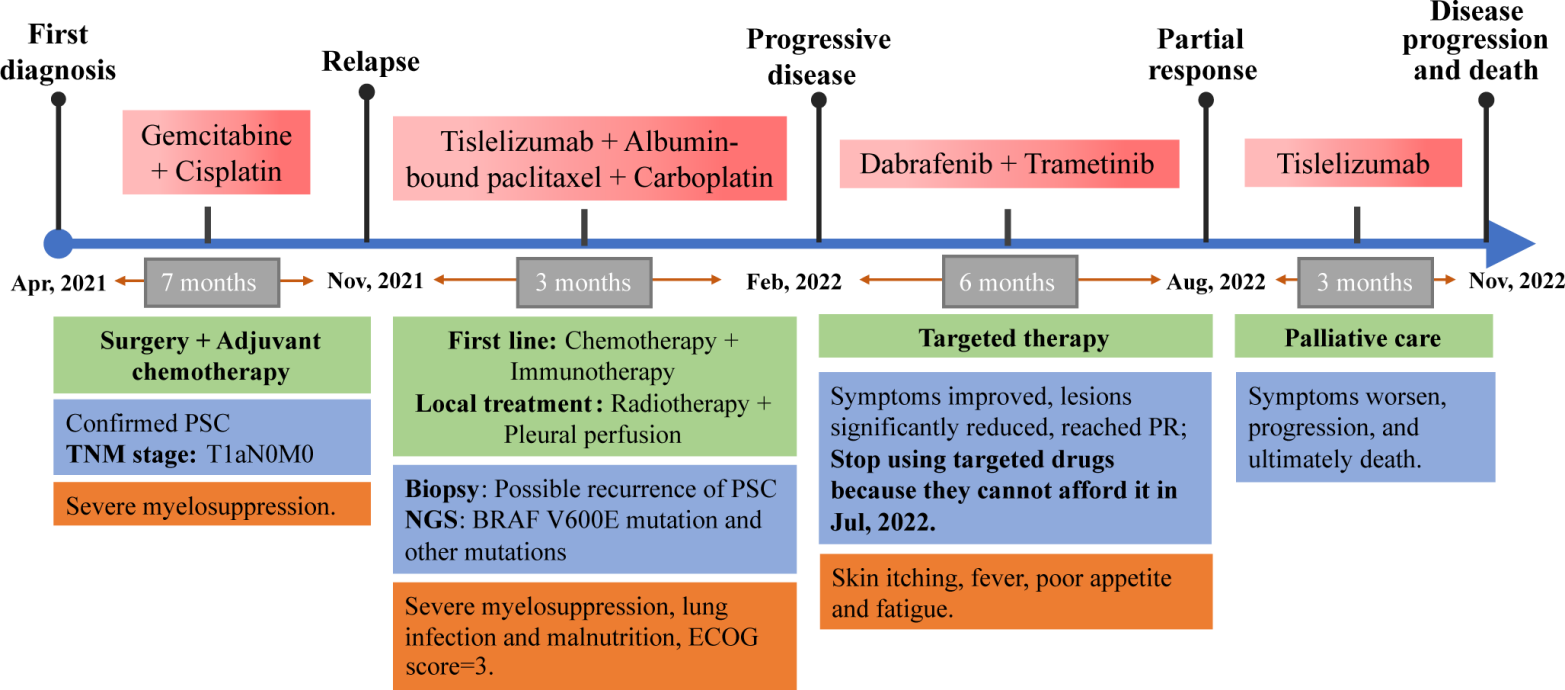
Complete diagnosis and treatment of this PSC patient. This patient with PSC had a rapid relapse following surgery and adjuvant chemotherapy in the early stages of the disease. NGS suggested that he had a BRAF^V600E^ mutation, but he refused targeted therapy for financial reasons, so chemotherapy combined with immunotherapy was used. However, the disease progressed rapidly, and the patient’s general condition deteriorated sharply. Desperate, the patient tried targeted therapy, achieved remission and six months of PFS. But he stopped taking targeted drugs for financial reasons, the disease progressed and eventually died. Due to the need for text editing, the time axis in the image is not proportional to the length of time.

To our knowledge, this is the first case report describing a clinical response to dabrafenib combined with trametinib in a patient with PSC harboring BRAF^V600E^ mutation, which demonstrates BRAF is one of the main drive oncogenes of PSC and is sensitive matched targeted therapy (**Figure 4**). Besides, this patient’s ECOG score had reached 3 when using targeted therapy. Targeted therapy controlled patient tumor progression without severe side effects, suggesting that targeted therapy is also appropriate for patients with high ECOG scores. Large-scale NGS panels may be able to provide more treatment options for patients with PSC. Moreover, this patient also had KRAS^G12A^ mutation, PIK3CA^E707K^ mutation, and TP53^H179R^ mutation. In resected NSCLC patients, KRAS transition mutations are significantly poor prognostic factors (41). According to the study of Lococo et al., PSC patients with mutations have a worse prognosis, especially the KRAS mutations alone or in combination with TP53, which is associated with decreased survival probability and occurrence of local metastases at recurrence (42). PIK3CA mutations are also associated with a poorer postoperative prognosis for PSC (43). However, in another clinical trial, NSCLC with KRAS co-mutated with TP53 had a better response to immunotherapy (44). We do not know how these mutations work together over the course of the disease, and whether they contribute to this patient’s insensitivity to chemotherapy and immunotherapy. The development of targeted drugs with multi-gene targets may be able to benefit such patients.

There also were limitations in this case. The patient underwent surgery and post-operative adjuvant chemotherapy at another hospital, the details of which are not available to us to rule out potential causes of recurrence. The patient had too few biopsies to accurately measure PD-L1 expression. In addition, larger prospective clinical trials are needed to confirm the efficacy of dabrafenib in combination with trametinib in BRAF^V600E^ mutant PSC.

## Conclusion

Dabrafenib in combination with trametinib provides partial remission in patients with advanced PSC with the BRAF^V600E^ mutation, which is more effective than chemotherapy in combination with immunotherapy, and it is also applicable when the ECOG score is high. Large-scale NGS panels may be able to provide more treatment options for patients with PSC.

## Data availability statement

The original contributions presented in the study are included in the article/**Supplementary Material**. Further inquiries can be directed to the corresponding author.

## Ethics statement

Written informed consent was obtained from the participant/patient(s) for the publication of this case report.

## Author contributions

RF: visualization and writing - original draft. JG: visualization and writing - review & editing. ZL: supervision and writing - review & editing. All authors contributed to the article and approved the submitted version.
