# Supplementary material for: An advanced pulmonary sarcomatoid carcinoma patient harboring a BRAFV600E mutation responds to dabrafenib and trametinib: a case report and literature review
Source: Front Oncol. 2023 Jul 21;13:1220745. doi: 10.3389/fonc.2023.1220745 (PMC10403232; doi:10.3389/fonc.2023.1220745)
Supplement: Supplementary file 1 [file DataSheet_1.pdf]

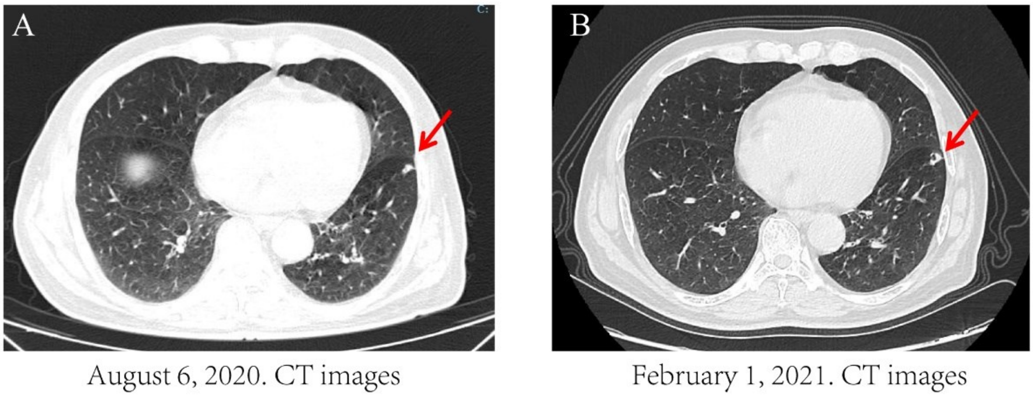

Fig S1 | The nodule in the left lower lung did not change significantly when the patient was initially presented with a cough. (A) CT image of the patient when he came to our hospital for chronic bronchitis in August 2020. The size of the nodules is 9 mm. (B) CT image of the patient when he came to our hospital with a cough. The size of the nodules is 10 mm.

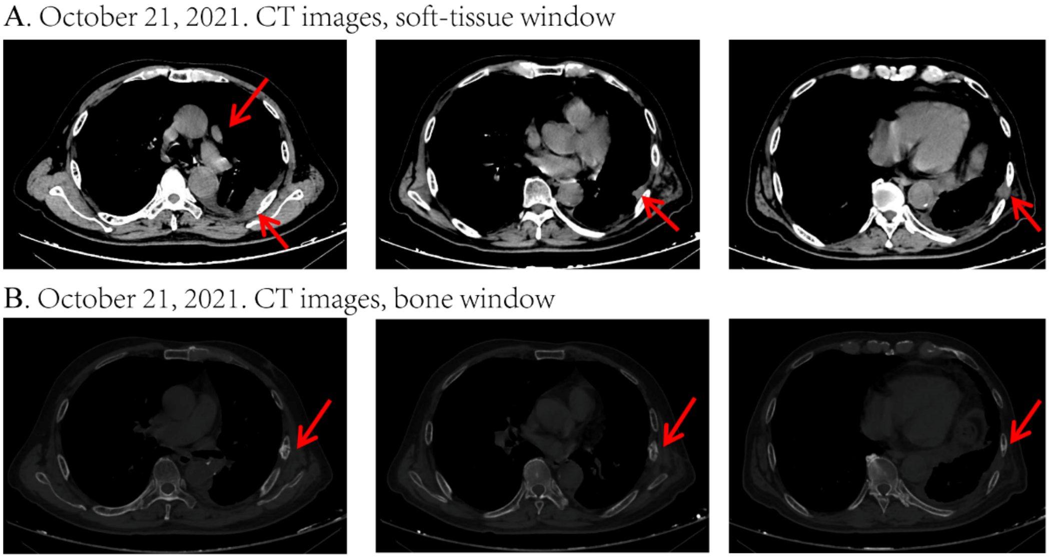

Fig S2 | CT image of the patient when he came to our hospital for chest pain in October 2021. (A) Enlarged mediastinal lymph nodes, left lung and subpleural nodules, and abnormally low-density shadows between left ribs can be seen in soft tissue window. (B) The bone window shows the destruction of the 6th, 7th, and 8th ribs on the left.

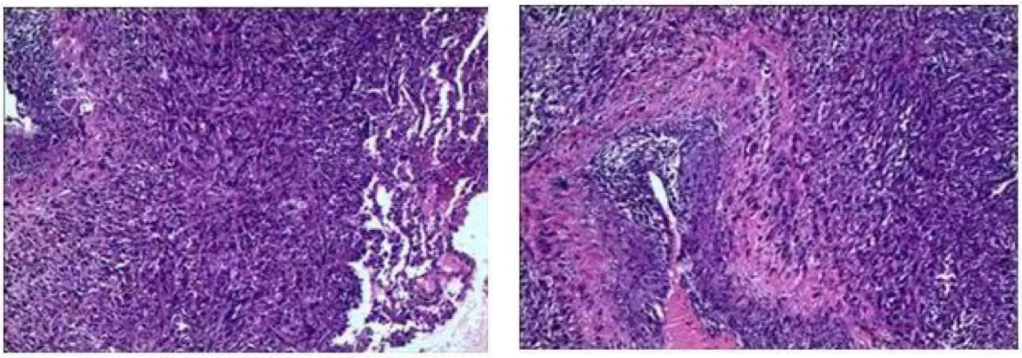

Fig S3 | Tissue images of patient's surgical specimen. A large number of spindle cells can be seen in the pathology images. Since the tissue images were obtained through pathology reports of patients from other hospital, the image quality is poor and is for reference only. Specific technical details are not available for the same reason. The immunohistochemical results were as follows: PCK (+), EMA (partial +), CK8/18 (+), CK7 (+), TTF-1 (partial +), NapsinA (partial +), CK5/6 (-), P63 (-), P40 (-), C-MET (-), ROS1 (partial +), P53 (medium-high strength +, consider mutant), Ki-67 (60%).

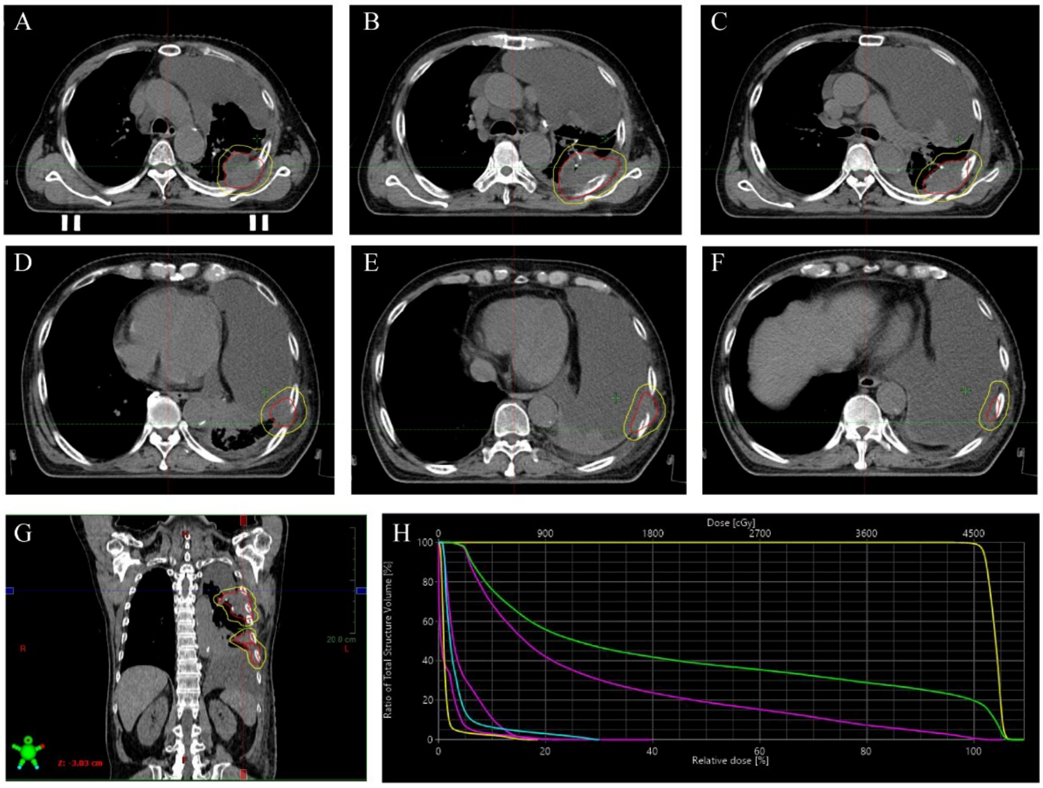

Fig S4 | Radiation therapy was administered to the patient to relieve the chest pain. (A-G) The radiotherapy target was set for the left damaged ribs and the surrounding carcinoma tissue. (H) Dose and volume histogram, intensity-modulated radiation therapy, Dt=45Gy/15F.

S5. Technical details of pathology

5.1 Immunohistochemistry

The automated platform: Leica BOND MAX

Table S1 | Antibody sources and information

Antibody	Corporation	Item No.	Clone No.
PD-L1	AmoyDx, China	-	E1L3N
CK7	ZSGB-BIO, China	ZM-0071	UMAB161
Napsin A	ZSGB-BIO, China	ZM-0473	OTI8A5
TTF-1	ZSGB-BIO, China	ZM-0270	SPT24
Vimentin	ZSGB-BIO, China	ZM-0260	UMAB159
CK	ZSGB-BIO, China	ZM-0069	AE1/AE3
CK5/6	ZSGB-BIO, China	ZM-0313	OTI1C7
P40	ZSGB-BIO, China	ZM-0472	BC28
Synaptophysin	ZSGB-BIO, China	ZM-0246	UMAB112
CD56	ZSGB-BIO, China	ZM-0057	UMAB83
Chromogranin A	ZSGB-BIO, China	ZM-0076	LK2H10
Ki-67	ZSGB-BIO, China	ZM-0167	MIB-1

5.2 Next-generation sequencing (NGS)

NGS platform: Life DA8600, proton

Table S2 | Gene panel

Project	Gene locus
Gene mutation	EGFR(NM_005228): Exon12, 18-21
	KRAS(NM_004985): Exon2-4
	BRAF(NM_004333): Exon12, 15
	PIK3CA(NM_06218.1): Exon10, 14, 21
	ERBB2(NM_004448): Exon19-21
	ERBB4(NM_005235): Exon3, 4, 6-9, 15, 23
	NRAS(NM_002524): Exon2-4
	PTEN(NM_000314.4): Exon1, 3, 6-8
	AKT1(ENST00000349310): Exon4
	MET(NM_000245): Exon2, 14, 16, 19
	CTNNB1(NM_001904): Exon32
	DDR2(NM_006182): Exon5, 8, 12-14

	ALK(NM_004304): Exon22, 23, 25	
	FBXW7(NM_033632.1): Exon5, 8-11	
	FGFR1(NM_000604): Exon4, 5, 7	
	FGFR2(NM_000141.2): Exon7, 9, 12	
	TP53(NM_000546): Exon4-8, 10	
	FGFR3(NM_000142): Exon7, 9, 14, 16, 18	
	MAP2K1(ENST00000307102): Exon5	
	NOTCH(NM_017617.2): Exon26, 27	
	STK11(NM_000455): Exon1, 4, 6, 8	
	SMAD4(NM_005359.3): Exon3, 5, 68-12	
Gene fusion	ALK(NM_004304.4)	ROS1(NM_002944.2)
	RET(NM_020975.4)	MET(NM_000245)
	NTRK1(ENST00000392302)	

**For financial reasons, this patient did not undergo a large-panel NGS, but routine genetic testing still provided effective treatment guidance information.*
